# Pancreatic schwannoma: a case report and an updated 40-year review of the literature yielding 68 cases

**DOI:** 10.1186/s12885-017-3856-6

**Published:** 2017-12-14

**Authors:** Yuntong Ma, Bingqi Shen, Yingmei Jia, Yanji Luo, Yisu Tian, Zhi Dong, Wei Chen, Zi-Ping Li, Shi-Ting Feng

**Affiliations:** 1 Department of Radiology, The First Affiliated Hospital, Sun Yat-Sen University, 58 Zhongshan 2nd Rd, Guangzhou, Guangdong 510080 China; 2 Washington University in St. Louis School of Medicine, St. Louis, MO USA; 3 Department of Pancreaticobiliary Surgery, The First Affiliated Hospital, Sun Yat-Sen University, Guangzhou, Guangdong China

## Abstract

**Background:**

Pancreatic schwannoma is a rare tumor. Preoperative diagnosis of pancreatic schwannoma is challenging due to its tendency to mimic other lesions of the pancreas. We describe a case of pancreatic schwannoma and present a review of the cases currently reported in the English literature to identify characteristics of pancreatic schwannoma on imaging.

**Case presentation:**

A 53-year-old male presented with a history of intermittent periumbilical abdominal pain and lower back pain for 1 week. Based on ultrasound (US) and computed tomography (CT) findings, we made a preoperative diagnosis of solid pseudopapillary tumor and performed a standard pancreaticoduodenectomy. Pathological examination showed that the tumor was composed of spindle cells with a palisading arrangement, and immunohistochemistry revealed strong positive staining for S-100 protein, which was consistent with a diagnosis of pancreatic schwannoma. At the 8-month follow-up visit, the patient was doing well without recurrent disease, and his abdominal pain had resolved.

**Conclusions:**

Although pancreatic schwannoma is rare, it should be included in the list of differential diagnoses of pancreatic masses, both solid and cystic. A tumor size larger than 6.90 cm, vascular encasement, or visceral invasion should elicit suspicion of malignant transformation.

## Background

Schwannomas, also known as neurilemmomas, are neoplasms arising from the Schwann cells of peripheral nerve sheaths [1, 2]. Schwannomas most frequently involve the head and neck area, major nerve trunks, and flexor aspects of the extremities. Deeply situated schwannomas are predominantly found in the retroperitoneum and posterior mediastinum but are rarely found in the trunk and gastrointestinal tract [1]. Pancreatic schwannomas are an extremely unusual variant of this neoplasm. According to a PubMed database search, 67 cases of pancreatic schwannomas have been described in the English literature over the past 40 years [3–64].

It has been reported that degenerative changes, such as cyst formation, hemorrhage, calcification, hyalinization and xanthomatous infiltration, are found in approximately two-thirds of pancreatic schwannomas [5, 6]. Degenerative changes lead to the presence of obvious variety in the appearance and size of the tumors. Preoperative diagnosis of pancreatic schwannoma can be particularly challenging. Pancreatic schwannomas may mimic other, more common pancreatic lesions, such as cystic neoplasms, solid and pseudopapillary neoplasms, pseudocysts and neuroendocrine tumors. Therefore, pancreatic schwannomas have a very high rate of misdiagnosis. Furthermore, schwannomas are benign peripheral nerve sheath tumors (PNSTs) by strict definition. However, they can undergo malignant degeneration, in which case they are called malignant PNSTs (MPNSTs) [6–11]. Simple enucleation is usually sufficient for benign pancreatic schwannomas, while extensive radical resection is recommended for patients with malignant tumors. However, how to distinguish malignant from benign pancreatic schwannomas remains challenging.

Consequently, a clearer consensus on the characteristics of pancreatic schwannomas is needed. For this purpose, we present herein a case of pancreatic schwannoma in a 53-year-old male and a review of the previous literature with an emphasis on radiographic features that may help distinguish between benign and malignant tumors.

## Case presentation

A 53-year-old male presented to our hospital in June 2016 with a history of intermittent periumbilical abdominal pain and lower back pain for 1 week. He did not exhibit any nausea/vomiting, jaundice or weight loss. Upon physical examination, the abdomen was soft and non-distended without evidence of hepatosplenomegaly or a palpable mass. His family history was not significant, and tumor markers (AFP, CA-125, CA19–9, and CEA) were within normal ranges.

**Fig. 1:**
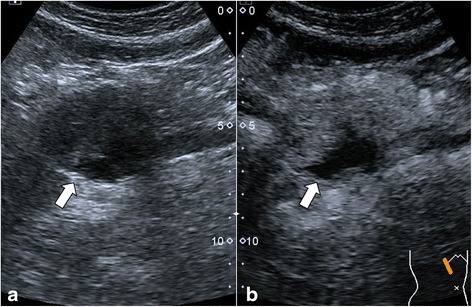
Abdominal US. **a**: Linear endoscopic ultrasound demonstrating a solid-cystic, heterogeneous, well-defined hypoechoic lesion in the head process of the pancreas (arrow). **b**: Early contrast-enhanced ultrasound showing an echogenic peripheral zone and a hypoechoic central area compared with the surrounding pancreatic parenchyma

**Fig. 2:**
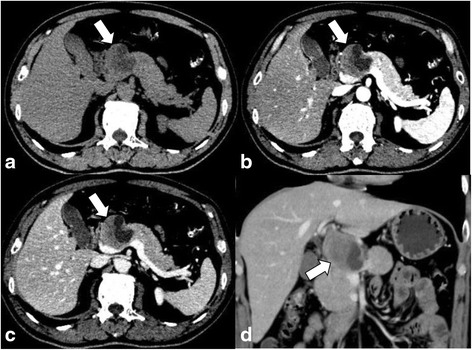
Computed tomography findings. **a**: An unenhanced CT scan showed a 4.8 × 4.6 cm well-defined cystic and solid mass (arrow) in the pancreatic head and adjacent to the portal vein. **b**: Enhanced CT scan revealed a mildly enhanced mass in the arterial phase (arrow). **c**: A moderately enhanced mass in the portal phase (arrow). **d**: A cystic and solid mass on coronal reconstruction (MPR) imaging

An abdominal US revealed a well-defined, hypoechoic mass in the head of the pancreas that measured 4.8 × 4.7 cm and contained an internal cystic component. On contrast-enhanced US, there was uniform enhancement of the tumor, and no intratumoral vascularity was detected with Doppler imaging (Fig. 1). Unenhanced computed tomography (CT) demonstrated a well-encapsulated, heterogeneous lesion at the junction of the pancreatic head and neck without calcification. On contrast-enhanced CT, there was mild and heterogeneous enhancement in the solid component of the tumor, which was less than the surrounding pancreatic parenchyma. The internal cystic component was not enhanced (Fig. 2). The mass compressed nearby blood vessels, such as the portal vein and celiac trunk, without invading them. No associated dilatation of the main pancreatic duct or common bile duct was found. Additionally, there were no liver masses or pathologic lymphadenopathy.

According to these results, the mass was preliminarily considered as a solid pseudopapillary tumor of the pancreas. The patient underwent exploratory laparotomy, which disclosed a well-encapsulated 6 × 5 cm mass arising from the head of the pancreas and compression of the portal and superior mesenteric veins. A standard Whipple pancreaticoduodenectomy was performed.

**Fig. 3:**
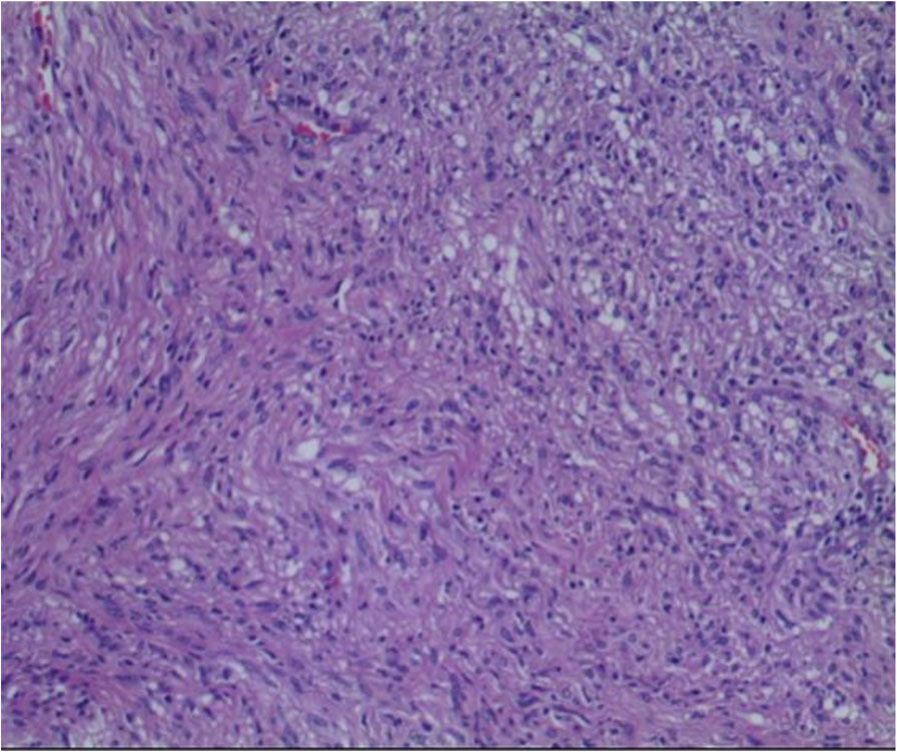
Microscopic examination. This tumor was mainly composed of spindle-shaped cells with palisading arrangement and no atypia, which is consistent with a benign schwannoma

Histopathological examination of the resected specimen revealed a 5.5 cm, well-circumscribed, yellow-gray mass with a slightly hard consistency. Microscopically, the tumor was completely surrounded by a capsule with subcapsular lymphocytic infiltrates and abundant lymphoid follicle formation. The tumor was composed of spindle cells with wavy nuclei and mild atypia exhibiting a palisading arrangement. Nerve fiber bundles were noted at the periphery (Fig. 3). Immunohistochemical staining results revealed that the tumor was strongly positive for CD-56 and S-100 but negative for CD-117, CD-34, DOG-1, desmin or smooth muscle actin. These findings were consistent with the final diagnosis of pancreatic schwannoma.

Postoperatively, the patient recovered well, and he was discharged without incident 3 weeks after the operation. At the 8-month follow-up visit, the patient was doing well without recurrent disease, and his abdominal pain had resolved.

## Discussion

Schwannomas, which were first described by Verocay in 1910, are mesenchymal neoplasms derived from Schwann cells that line peripheral nerve sheaths and do not contain neuroganglion cells [65]. However, the pancreas is an extremely uncommon site of origin for schwannomas, which arise from either the sympathetic or parasympathetic nerve fibers coursing through the pancreas [29]. A literature search was performed in March 2017. The MeSH term used in the PubMed search was ‘pancreatic schwannoma’. In addition, the reference lists of relevant articles were searched to find other eligible studies. A PubMed search of the past four decades revealed 62 articles describing 67 cases of pancreatic schwannoma in the English literature [3–64]. A total of 68 cases of pancreatic schwannoma, including our current case, were analyzed and summarized. The clinicopathological data for all 68 patients with pancreatic schwannoma was shown in Table 1. All of the data were analyzed with SPSS 17.0 software (Chicago, IL, USA). Continuous variables were recorded as the means ± standard deviation (SD) and range and were analyzed using a two-tailed Student’s t-test. The tumor distribution was skewed in the control group, so the Wilcoxon rank sum test of two independent samples was used. Categorical variables were analyzed with the four-fold table method and the R*2 method of the Chi-squared test. *P* values <0.05 were considered statistically significant. Receiver-operating characteristic (ROC) curves were used to determine the optimal cut-off points of tumor size for distinguishing malignant lesions from benign lesions.

**Table 1:** Summary of clinicopathological data from all 68 cases of pancreatic schwannoma [3–64]

	*n* (%) or mean ± SD (range)
Age (years) (*n* = 68)	
Mean	55.7 ± 14.7 (20–87)
Sex (male/female), (male %) (*n* = 68)	15:19 (44%)
Symptoms (*n* = 68)	
Asymptomatic	23 (34%)
Symptoms	
Abdominal pain	34 (50%)
Weight loss	12 (18%)
Nausea/vomiting	6 (8.8%)
Dyspepsia	5 (7.4%)
Back pain	4 (5.9%)
Abdominal mass	3 (4.4%)
Anemia	2 (2.9%)
Melena	2 (2.9%)
Jaundice	2 (2.9%)
Mean size (cm) (*n* = 64)	6.1 ± 5.7 (1–33)
Location (*n* = 67)	
Uncinate	9 (13%)
Head	27 (40%)
Head + body	3 (4.5%)
Body	14 (21%)
Body + tail	7 (10%)
Tail	6 (9.0%)
Nature of tumor on imaging (*n* = 68)	
Solid	19 (28%)
Cystic	29 (43%)
Solid + cystic	15 (22%)
Not specified	5 (7.4%)
Imaging characteristics	
Well-defined margins (*n* = 59)	47 (80%)
Presence of calcifications (*n* = 58)	5 (8.6%)
Pancreatic duct/CBD dilation (*n* = 22)	2 (9.1%)
Relation to nearby structures (*n* = 29)	
No changes to nearby structures	12 (41%)
Compressed/displaced vessels	9 (31%)
Encasement of vessels	4 (14%)
Invasion of viscera	3 (10%)
Preoperative diagnosis (*n* = 44)	
Correct	9 (20%)
Incorrect	35 (80%)
Cystic neoplasm	17 (49%)
Serous cystic neoplasm	3 (8.6%)
Mucinous cystic neoplasm	8 (23%)
Solid pseudopapillary neoplasm	7 (20%)
Pancreatic neuroendocrine tumor	8 (23%)
Pseudocyst	2 (5.7%)
Acinar cell carcinoma	3 (8.6%)
Mucinous cystadenocarcinoma	6 (17%)
Treatment (*n* = 67)	
Pancreaticoduodenectomy	16 (24%)
Pylorus-preserving pancreaticoduodenectomy	7 (10%)
Central pancreatectomy	2 (3.0%)
Distal pancreatectomy +/− splenectomy	15 (22%)
Enucleation	8 (12%)
Surgical resection not otherwise specified (NOS)	14 (21%)
Unresectable	2 (3.0%)
Refused	3 (4.5%)
Histology (*n* = 68)	
Benign	60 (88%)
Malignant	8 (12%)
Mean follow-up (months) (*n* = 34)	19 ± 15.4 (3–65)
Died of disease	1

Pancreatic schwannomas may develop in any part of the pancreas but are most commonly located in the pancreatic head. Overall, approximately one-third of patients are asymptomatic. Common symptoms are abdominal pain and weight loss, while a few patients present with nausea, vomiting, dyspepsia, back pain or a palpable mass. Anemia, melena and jaundice are infrequent symptoms. Laboratory tests, including tumor markers, are usually within the normal range. Pancreatic schwannomas are usually benign and solitary. However, there may be multiple malignant schwannomas in patients with von Recklinghausen disease (VRD) [61]. In the 68 cases in this review, only 2 patients reported a history of VRD [7, 11].

Definitive diagnosis is achieved only with histological examination and immunohistochemical staining of a surgically resected specimen. Macroscopically, pancreatic schwannomas are well-circumscribed, encapsulated, homogeneous, yellow-tan nodules. Up to two-thirds of pancreatic schwannomas exhibit secondary degenerative changes, such as cyst formation, hemorrhage, hyalinization, calcification and xanthomatous infiltration [26, 37, 62]. Microscopically, typical features of pancreatic schwannomas include an organized hypercellular component (Antoni A areas) and a hypocellular component with loose myxoid stroma (Antoni B areas). Antoni B areas often have accompanying degenerative changes [4]. Benign schwannomas normally have < 5 mitotic figures per 10 high-powered fields [58]. Pancreatic schwannomas demonstrate strong positive immunohistochemical staining for S-100, vimentin, and CD-56, and they show negative staining for cytokeratin AE1/AE3, CD34, CD117 (c-kit), desmin, and smooth muscle myosin [66].

An accurate preoperative diagnosis of pancreatic schwannomas is difficult due to the nonspecific radiological appearance of these tumors, even with the use of multiple imaging modalities. Due to the variable proportions of Antoni A and B areas contained in the tumor, pancreatic schwannomas commonly appear cystic (near half of cases, 29; 43%) but may also have a completely solid structure (19; 28%) or a mixed solid and cystic pattern (15; 22%) (Table 1). Up to 80% of schwannomas exhibited well-defined margins on imaging and were also found to be well encapsulated upon gross pathological examination (Table 1). On ultrasound, a pancreatic schwannoma usually appears as a well-defined, hypoechoic lesion that shows poor contrast intake and is hypoenhanced compared to the surrounding pancreatic parenchyma [22, 44]. On unenhanced CT, tumors composed of exclusively or predominantly Antoni A areas appear as well-defined, heterogeneous, hypodense, solid masses due to their high cellularity and increased lipid content, whereas tumors with high Antoni B areas appear as homogeneous cystic masses due to their low cellularity and loose myxoid stroma [56]. Contrast-enhanced CT is helpful for distinguishing between Antoni A and Antoni B areas based on their vascularity. Generally, the more vascular Antoni A areas are enhanced, whereas Antoni B areas are frequently not enhanced [56, 64]. On magnetic resonance imaging (MRI), schwannomas appear homogeneously hypointense on T1-weighted imaging (T1WI) and hyperintense on T2-weighted imaging (T2WI) [45]. Sometimes, they may also appear inhomogeneously intense due to the presence of a fair amount of both Antoni A and Antoni B areas in the tumors [56]. Calcifications (5; 8.6%) and associated pancreatic ducts or common bile duct dilation (2; 9.1%) are uncommon findings but are both associated with benign tumors. Larger tumors (9; 31%) may cause the compression or displacement of nearby vessels. More rarely, tumors (4; 14%) are found to completely encase adjacent vessels or invade the duodenum and transverse colon (Table 1). Lymph node enlargement and distant metastases are typically not associated with pancreatic schwannomas. Due to the variable radiographic appearance of schwannomas, they often mimic other pancreatic tumors that share similar imaging features, leading to a high rate of misdiagnosis. The most common differential diagnosis is cystic neoplasms, such as serous or mucinous cystic neoplasms. Pancreatic schwannomas are also misdiagnosed as pancreatic neuroendocrine tumors, solid pseudopapillary tumors, mucinous cystadenocarcinomas, acinar cell carcinomas and pancreatic pseudocysts. Endoscopic ultrasound-guided fine needle aspiration (EUS-FNA) may contribute to precise preoperative diagnosis, but its use remains controversial because of its high false-negative rate. In the 16 patients with EUS-FNA, only 8 (50%) were correctly diagnosed preoperatively with pancreatic schwannoma.

**Fig. 4:**
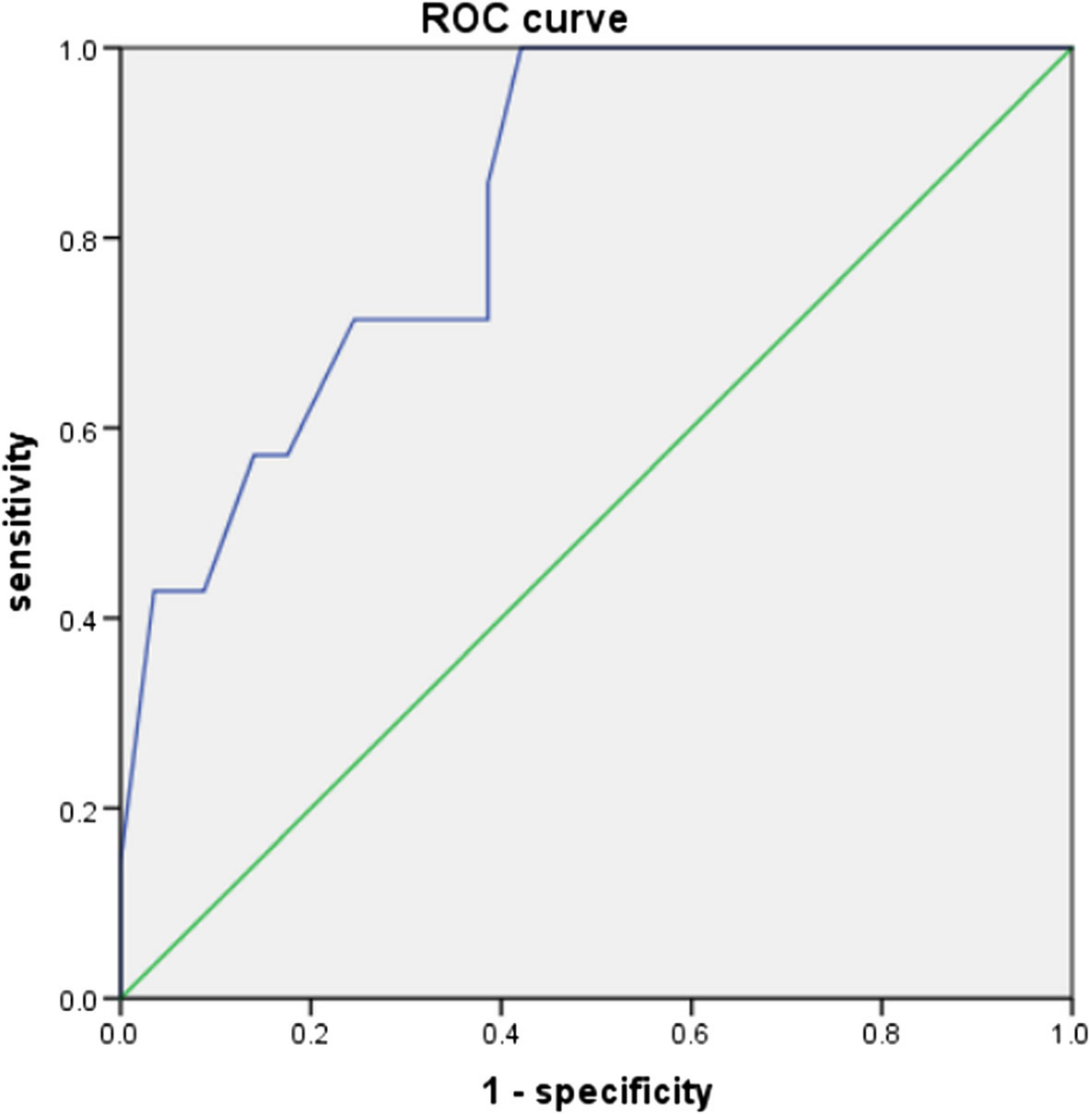
ROC curve for tumor size to distinguish malignant pancreatic schwannoma from benign tumors (area under the curve: 0.836, cut-off: 6.90 cm, sensitivity: 71.4%, specificity: 75.4%)

The vast majority of pancreatic schwannomas (approximately 88.2%) are benign [3]. Malignant transformation is extremely rare. Only 8 cases (11.8%) of malignant pancreatic schwannoma have been described in the English literature [6–11]. Some investigators have attempted to correlate the tumor’s characteristics on imaging with its malignant potential. Moriya et al. [4] reported that larger tumors correlated with greater malignant potential. This was confirmed in our current analysis of an updated dataset. The characteristics of benign and malignant pancreatic schwannomas were summarized in Table 2. Pancreatic schwannomas had a mean size of 5.2 ± 4.0 cm for benign tumors and 14.0 ± 10.3 cm for malignant tumors, a difference that was statistically significant. A ROC curve of tumor size was constructed with an area under the curve calculated as 0.836, a cut-off of 6.90 cm, a sensitivity of 71.4% and a specificity of 75.4% (Fig. 4). We further analyzed other tumor characteristics on imaging to determine the predictors of malignancy on pathological examination. However, there was no correlation between whether tumors appeared to be solid, cystic, or have a mixed pattern as a predictor of malignancy (Table 2). Benign tumors were significantly more likely than malignant ones to exhibit well-defined margins on imaging, whereas the presence of calcifications and pancreatic duct or common bile duct dilation was not significant. However, the presence of well-defined margins did not exclude malignancy because 2 cases of malignant schwannoma were also initially described as having well-defined radiographic margins. Moreover, malignant tumors were more likely than benign tumors to cause changes in adjacent structures, including the encasement of nearby vessels, such as the portal vessels, celiac trunk, and superior mesenteric vessels, as well as the invasion of adjacent organs, such as the small and large bowel on surgical or pathological examination (Table 2).

**Table 2:** Comparison of characteristics between benign and malignant pancreatic schwannomas

	Benign (*n* = 60)	Malignant (*n* = 8)	*P*
Mean age (years)	56.3 ± 14.4 (20–87)	51.1 ± 17.7 (24–74)	0.358
Sex (male/female), (male %)	9:11 (45%)	3:5 (37.5%)	1.000
Mean size (cm)	5.2 ± 4.0 (1–20)	14.0 ± 10.3 (5–33)	0.004
Location			
Uncinate	9 (15%)	0 (0%)	0.228
Head	22 (37%)	5 (71%)	
Head + body	3 (5%)	0 (0%)	
Body	14 (23%)	0 (0%)	
Body + tail	6 (10%)	2 (29%)	
Tail	6 (10%)	0 (0%)	
Nature of tumor on imaging			
Solid	17 (30%)	2 (25%)	0.845
Cystic	26 (46%)	3 (37.5%)	
Solid + cystic	13 (23%)	2 (25%)	
Not specified	4 (7.1%)	1 (12.5%)	
Imaging characteristics			
Well-defined margins	45 (96%)	2 (33%)	0.009
Presence of calcifications	5 (9.4%)	0 (0%)	1.000
Pancreatic duct/CBD dilation	2 (9.1%)	0 (0%)	1.000
Relation to nearby structures			
No changes in nearby structures	12 (52%)	0 (0%)	< 0.001
Compressed/displaced vessels	9 (39%)	0 (0%)	
Encasement of vessels	2 (8.7%)	3 (50%)	
Invasion of viscera	0 (0%)	3 (50%)	
Treatment			
Pancreaticoduodenectomy	13 (22%)	3 (37.5%)	
Pylorus-preserving pancreaticoduodenectomy	7 (10%)	0 (0%)	
Central pancreatectomy	2 (3.0%)	0 (0%)	
Distal pancreatectomy +/− splenectomy	13 (19%)	1 (12.5%)	
Enucleation	8 (12%)	0 (0%)	
Surgical resection NOS	13 (19%)	2 (25%)	
Unresectable	0 (0%)	2 (25%)	
Refused	3 (4.5%)	0 (0%)	
Mean follow-up (months)	19.4 ± 16.0 (3–65)	16.3 ± 11.6 (4–28)	
Died of disease	0	1

The management of pancreatic schwannomas is guided by location and histological results. Because most of these tumors are benign and malignant transformation is rare, simple enucleation is usually sufficient if pathology is confirmed before surgery [63]. In cases of large tumors, especially those exhibiting malignant behavior (infiltration of tissue), the presence of intangibility in frozen section or close proximity to major vessels, an oncological margin-negative resection is recommended [6]. In a review of treatments, the most common type of surgical resection was pancreaticoduodenectomy, followed by distal pancreatectomy with/without splenectomy, enucleation, pylorus-preserving pancreaticoduodenectomy, and central pancreatectomy. The high frequency of extensive radical resection may reflect the difficulties in obtaining an accurate preoperative diagnosis of pancreatic schwannomas and in distinguishing these lesions from other pancreatic neoplasms. In such cases, intraoperative frozen sections should be performed to help establish the diagnosis of a benign schwannoma and avoid more extensive resection [4]. Following tumor excision, the long-term prognosis of pancreatic schwannoma is excellent, with no cases of recurrence over a mean follow-up of 19 ± 15.4 months (range 3–65 months).

## Conclusions

Although pancreatic schwannoma is rare, it should be included in the list of differential diagnoses of pancreatic masses, both solid and cystic. A tumor size larger than 6.90 cm, vascular encasement, or visceral invasion should elicit suspicion of malignant transformation.

## Abbreviations

AFP: Alpha fetoprotein
CA-125: Carbohydrate antigen 125
CA19–9: Carbohydrate antigen 19–9
CD: Cluster of differentiation
CEA: Carcinoembryonic antigen
CT: Computed tomography
DOG-1: Discovered on GIST-1
EUS-FNA: Endoscopic ultrasound-guided fine needle aspiration
GIST: Gastrointestinal stromal tumor
MRI: Magnetic resonance imaging
PNSTs: Peripheral nerve sheath tumors
ROC curves: Receiver-operating characteristic curves
SD: Standard deviation
T1WI: T1-weighted imaging
T2WI: T2-weighted imaging
US: Ultrasound
VRD: Von Recklinghausen disease
